# Iodine supplementation: compliance and association with adverse obstetric and neonatal outcomes

**DOI:** 10.1530/ETJ-21-0035

**Published:** 2021-09-16

**Authors:** Maria Lopes-Pereira, Anna Quialheiro, Patrício Costa, Susana Roque, Nadine Correia Santos, Margarida Correia-Neves, Ana Goios, Ivone Carvalho, Tim I M Korevaar, Laura Vilarinho, Joana Almeida Palha

**Affiliations:** 1 Life and Health Sciences Research Institute (ICVS), School of Medicine, University of Minho, Braga, Portugal; 2 ICVS/3B’s, PT Government Associate Laboratory, Braga/Guimarães, Portugal; 3 Hospital de Braga, Braga, Portugal; 4 ACMP5 – Associação Centro de Medicina P5 (P5), School of Medicine, University of Minho, Braga, Portugal; 5 Newborn Screening, Metabolism & Genetics Unit, National Institute of Health Dr Ricardo Jorge, Porto, Portugal; 6 Academic Center for Thyroid Diseases, Erasmus MC, Rotterdam, the Netherlands; 7 Department of Internal Medicine, Erasmus MC, Rotterdam, the Netherlands; 8 The Generation R Study Group, Erasmus MC, University Medical Center, Rotterdam, the Netherlands; 9 Clinical Academic Center-Braga (2CA-B), Braga, Portugal

**Keywords:** iodine, pregnancy, newborn, obstetric outcomes, neonatal outcomes

## Abstract

**Objectives:**

Over 1.9 billion people worldwide are living in areas estimated to be iodine insufficient. Strategies for iodine supplementation include campaigns targeting vulnerable groups, such as women in pre-conception, pregnancy and lactation. Portuguese women of childbearing age and pregnant women were shown to be mildly-to-moderately iodine deficient. As a response, in 2013, the National Health Authority (NHA) issued a recommendation that all women considering pregnancy, pregnant or breastfeeding, take a daily supplement of 150–200 μg iodine. This study explored how the iodine supplementation recommendation has been fulfilled among pregnant and lactating women in Portugal, and whether the reported iodine supplements intake impacted on adverse obstetric and neonatal outcomes.

**Design and methods:**

Observational retrospective study on pregnant women who delivered or had a fetal loss in the Braga Hospital and had their pregnancies followed in Family Health Units.

**Results:**

The use of iodine supplements increased from 25% before the recommendation to 81% after the recommendation. This was mostly due to an increase in the use of supplements containing iodine only. Iodine supplementation was protective for the number of adverse obstetric outcomes (odds ratio (OR) = 0.791, *P* = 0.018) and for neonatal morbidities (OR = 0.528, *P* = 0.024) after controlling for relevant confounding variables.

**Conclusion:**

The recommendation seems to have succeeded in implementing iodine supplementation during pregnancy. National prospective studies are now needed to evaluate the impact of iodine supplementation on maternal thyroid homeostasis and offspring psychomotor development and on whether the time of the beginning of iodine supplementation (how early during preconception or pregnancy) is relevant to consider.

## Introduction

Iodine is an essential nutrient required for the biosynthesis of thyroid hormone. Thyroid hormone regulates growth and metabolism and is essential for proper fetal brain- and nervous system development (1). During pregnancy iodine requirements are higher, due to the growing fetus and various physiological adjustments (2), which renders pregnant women more susceptible to iodine insufficiency. Of concern, several studies found an association of iodine insufficiency with increased maternal thyroid volume and structure, adverse pregnancy outcomes, perinatal and infant mortality (3, 4, 5, 6, 7), and altered psychomotor development of the newborn, with consequences extended further on during childhood (8, 9). Although the deleterious effects of severe iodine deficiency are well recognized, the benefits of interventions correcting mild-to-moderate iodine deficiency are uncertain. The latest Cochrane review showed that evidence is still insufficient to conclude on the benefits and harms of iodine supplementation, and that further well-designed studies are necessary (10).

In 2009, we (11) and others (12) showed that Portuguese women of childbearing age and pregnant women had insufficient iodine intake and that pregnant women in the Minho region of Portugal lacked the physiological thyroid hormone adaptations typically observed in otherwise healthy pregnant women from iodine-sufficient populations (13). Moreover, during follow-up, we observed that maternal serum free thyroxine levels in the 1st trimester of pregnancy predicted infant psychomotor development, with altered behavior already detected at 12 months (14). These findings contributed to support a recommendation of the Portuguese National Health Authorities (NHA), in 2013, to provide iodine supplementation (150–200 μg/day) to women during preconception, pregnancy and lactation (15).

Eight years past this recommendation, the present retrospective study was undertaken to explore how the iodine supplementation recommendation has been fulfilled and whether the reported use of iodine supplements was associated with obstetrics and neonatal outcomes.

## Materials and methods

### Study design and population

This observational retrospective study was conducted in Braga (the regional capital of the Minho region in Northern Portugal) in 10 out of 17 family health units (USF, Unidade de Saúde Familiar, in Portuguese) of the grouping health centers of Cávado I (ACES Cávado I). These 10 USF were selected as those contributing the most to the total number of births in Braga Hospital between 2013 and 2017 (65%).

The inclusion criteria were pregnant women who delivered or had a fetal loss in the Braga Hospital and had their pregnancies followed up until birth in the selected USFs.

Since the Portuguese National Health Authority (NHA) recommendation on iodine supplementation was published in August 2013, the study sample was analyzed in two groups: (1) before the recommendation group (BR), including those fulfilling the inclusion criteria and who delivered between January and September 2013; and (2) after the recommendation group (AR), including those who fulfilled the inclusion criteria and delivered between July 2014 and December 2017. Births before 2013 were not included due to scarcity of clinical registers.

**Figure 1:**
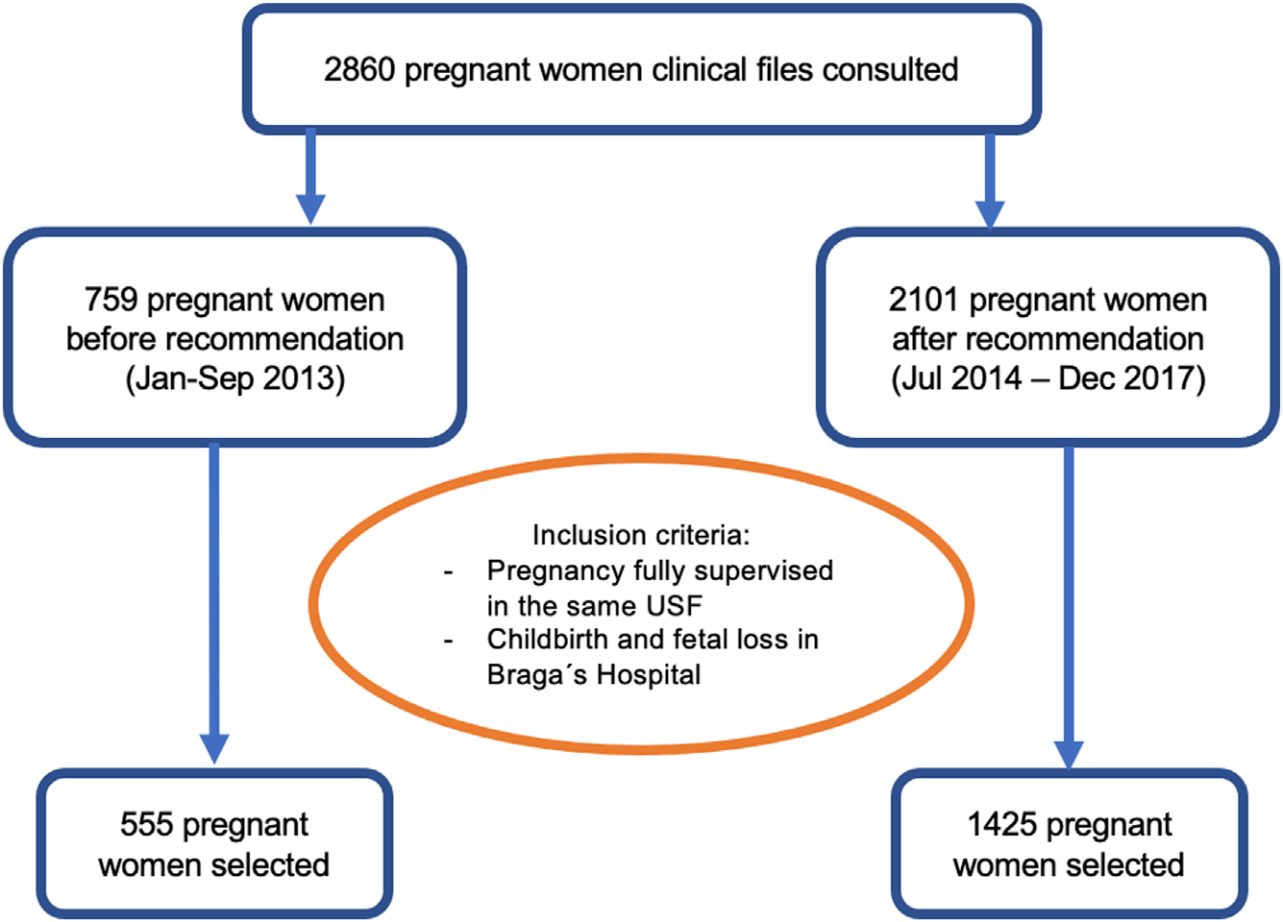
Study flowchart.

From the period under study, we collected data from 2860 pregnant women of which 1980 (69%) fulfilled the inclusion criteria (555 BR and 1425 AR) (Fig. 1).

The manuscript was written respecting the Strengthening the Reporting of Observational Studies in Epidemiology (STROBE) checklist.

### Data collection

Clinical and socioeconomic data regarding background variables and adverse obstetric and neonatal outcomes were retrieved from obstetrics clinical registers from the Department of Obstetrics of Braga’s Hospital and from the USF’s Maternal Health Consultation. Data collected included age, level of education, parity, previous miscarriages, smoking and alcohol consumption, records of thyroid disease and obesity, height, weight, preconception BMI (kg/m^2^) and gestational gain weight, reported iron and folic acid supplementation during pregnancy, reported iodine supplementation (since, in most cases, no information was available on the precise start time, iodine supplementation may have started any time during preconception, pregnancy or lactation), gestational age at birth, mode of delivery (caesarean section/vaginal delivery/instrumental delivery), Apgar score and newborn birth weight, length and cephalic perimeter. Newborn TSH levels, between 3rd and 6th day of life, were obtained from the National Neonatal Screening Programme; normal values were considered if <10 mU/L (16).

Reported use of iodine supplements was the exposure variable; adverse obstetric outcomes (fetal loss (defined as miscarriage before 20 weeks and stillbirth after 20 weeks), preterm delivery (23–37 weeks), risk of preterm delivery, gestational hypertension and preeclampsia, gestational diabetes, hydramnios, intrauterine growth restriction, placental abruption, small or large for gestational age (defined as birth weight below the 10th and above the 90th percentile, respectively, for gestational age and sex (17)), and congenital malformations) and neonatal morbidities (admission to the neonatal care unit), the main outcome measures.

The following variables were considered as potential confounders: maternal age (increasing maternal age is independently associated with specific adverse pregnancy outcomes (18)), level of education (women with lower levels of education are at greater risk for adverse maternal outcomes (19)), parity (knowing that nulliparity has a higher risk of adverse pregnancy outcomes than low multiparity (20)), previous miscarriages (a previous miscarriage is associated with a higher risk of obstetric complications (21)), smoking (exposure to smoke during pregnancy is associated with an increased risk of obstetric complications and adverse health outcomes for children exposed *in utero* (22)), alcohol consumption (exposure to alcohol *in utero* can have numerous adverse effects on a developing fetus (23)), preconception BMI (maternal pre-pregnancy obesity has been associated with higher obstetric outcomes (24)), thyroid disease (overt hypothyroidism and overt hyperthyroidism are reported to increase the risk of miscarriage, preterm delivery and other adverse pregnancy outcomes (25)), and iron and folic supplementation (supplements with iron and folic acid are reported to protect from some neonatal morbidities (26)).

### Statistical analysis

Independence chi-square test was used for qualitative variables and unpaired samples *t*-test or Mann–Whitney test to compare maternal variables and newborn variables, considering intake/no intake of iodine supplementation and also for the comparisons of BR and AR. The tests assumptions were evaluated. For the chi-square test, a maximum of 20% of expected counts lower than 5 and none less than 1, for 2 by 2 tables Fisher's exact test were reported. Assumption of normality of the distribution was assessed visually through histograms, using the Kolmogorov–Smirnov and Shapiro–Wilk tests and the skewness and kurtosis measures. For variables with relevant deviations from a normal distribution, the non-parametric correspondent tests were applied, and medians and the interquartile range were reported.

Since women could have more than one adverse obstetric outcomes, Poisson regression was used to predict the number of occurrences. Binary logistic regression was used to predict neonatal morbidities.

For Poisson regression analysis, there was complete information for 1123 women (769 with iodine, 354 without iodine); this population was identical to the original, except for age, in which women with iodine supplementation were 1 year older on average (*P* = 0.02).

For binary logistic regression analysis, there was complete information for 1012 women (728 with iodine, 284 without iodine); this population was identical to the original in demographic and clinical variables, except for age, in which women with iodine supplementation are 1 year older on average (*P* = 0.009).

While folic acid intake would be a possible confounder for adverse obstetric and neonatal outcomes, it was not considered in the models since all women taking iodine also reported folic acid intake.

Multicollinearity was assessed through the tolerance values for all predictors; the minimum observed value was of 0.516 for education (having a higher education degree).

Statistical analyses were performed using IBM SPSS Statistics (version 25) (IBM Corp.) and results were considered significant when *P* < 0.05. Measures of effect sizes were calculated with the correspondent cut-off values: for Pearson’s ϕ, Cramer’s V and r, 0.1 small, 0.3 medium and 0.5 large; and for Cohen’s *d*, 0.2 small, 0.5 medium and 0.8 large (27).

## Results

**Figure 2:**
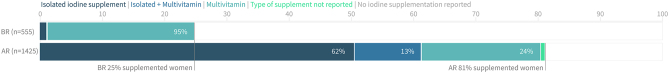
Percentage of women reporting iodine intake and the corresponding intake formula.

The proportion of women reported to use iodine supplements increased from 25% BR to 81% AR. The observed increase was mostly due to the use of isolated iodine pills (Fig. 2).

Sociodemographic and clinical information are presented in Table 1. Women in the iodine supplemented group had more years of formal education (small effect size, Pearson’s ϕ = 0.078). Less women in the iodine supplemented group had thyroid disease (small effect size, Cramer’s V = 0.116), smoked (small effect size, Pearson’s ϕ = 0.069) or consumed alcohol (small effect size, Pearson’s ϕ = 0.096). More women taking iodine supplements also reported folic acid (medium effect size, Pearson’s ϕ = 0.344) and iron supplementation (medium effect size, Pearson’s ϕ = 0.298). Parity, previous miscarriages, preconception BMI, previous obesity and weight gain did not differ among groups.

**Table 1:** Participant’s characterization^a^.

	No iodine, *n* = 687	With iodine, *n* = 1293	*P* value
Maternal age (years; mean ± s.d.)	32 ± 6	32 ± 5	0.299
Education (%)			
≤9 years	22	19	
10-12 years	33	28	
Certificate after 12th grade	45	53	0.007
Parity (%)			
Nullipara	48	52	
Multiparous (≥1)	52	48	0.093
Previous miscarriages (%)	21	20	0.526
Thyroid disease (%)	13	6	<0.001
Smoking (%)	20	15	0.004
Alcohol consumption (%)	12	7	<0.001
Folic acid supplementation (%)	84	100	<0.001
Iron supplemmentation (%)	71	93	<0.001
Previous obesity (%)	13	16	0.181
Preconception BMI (kg/m^2^; mean ± s.d.)	24.2 ± 4.5	24.1 ± 4.4	0.868
Weight gain (kg; mean ± s.d.)	12.4 ± 4.8	12.6 ± 5.0	0.647

^a^No information on: education (for 224 women with iodine supplementation and for 119 women without iodine supplementation), parity (for 4 women with iodine supplementation and for 1 woman without iodine supplementation), previous miscarriages (for 1 woman with iodine supplementation and for 1 woman without iodine supplementation), thyroid disease (for 12 women with iodine supplementation and for 17 women without iodine supplementation), smoking (for 106 women with iodine supplementation and for 84 women without iodine supplementation), alcohol consumption (for 124 women with iodine supplementation and for 85 women without iodine supplementation), folic acid supplementation (for 28 women with iodine supplementation and for 140 women without iodine supplementation), iron supplementation (for 133 women with iodine supplementation and for 193 women without iodine supplementation), previous obesity (for 535 women with iodine supplementation and for 167 women without iodine supplementation), preconception BMI (for 195 women with iodine supplementation and for 142 women without iodine supplementation), weight gain (for 457 women with iodine supplementation and for 343 women without iodine supplementation).

Table 2 provides information on the adverse obstetric outcomes. Considering all pregnancies, the number of women experiencing adverse obstetric outcomes was higher in the non-iodine supplemented group (small effect size, Pearson’s ϕ = 0.167), as were the number of miscarriages (small effect size, Pearson’s ϕ = 0.119). No differences were observed on the adverse obstetric outcomes from viable pregnancies. One hundred thirty-five women had more than one adverse obstetric outcome, with a maximum of 6.

**Table 2:** Adverse obstetric outcomes.

All pregnancies	No iodine, *n* = 687	With iodine, *n* = 1293	*P* value
Adverse obstetric outcomes (%)	50	33	<0.001
Miscarriages (%)	16	8	<0.001
Stillbirths (%)	0	0.4	0.171^a^
**Viable pregnancies**	No iodine supplementation, *n* = 479	Iodine supplementation, *n* = 1162	*P* **value**
Fetal malformations (%)	2	1	0.093
Gestational diabetes (%)	10	11	0.490
Gestational hypertension (%)	2	2	0.363
Hydramnios (%)	0.2	0.8	0.298^a^
Intrauterine growth restriction (%)	3	4	0.408
Large for gestational age (%)	3	3	0.993
Placental abruption (%)	0	0.4	0.330^a^
Preeclampsia (%)	0.6	0.9	0.769^a^
Preterm delivery (%)	5	4	0.113
Risk of preterm delivery (%)	3	3	0.927
Small for gestational age (%)	13	14	0.378

^a^Fisher’s test. Adverse obstetric outcomes (miscarriages, stillbirth, fetal malformations, gestational diabetes, gestational hypertension, hydramnios, intrauterine growth restriction, large for gestational age, placental abruption, preeclampsia, preterm delivery, risk of preterm delivery, small for gestational age).

The analysis can be further explored, considering the time before and after the recommendation (Supplementary Tables 1 and 2, see section on supplementary materials given at the end of this article).

Regression analysis allowed us to control for confounding with respect to the occurrence of adverse obstetric outcomes (Table 3). Intake of iodine and iron were associated with a 25% (even though not reaching statistical significance, *P* = 0.055) and 87% (*P* < 0.001) decreased odds, respectively, of having an adverse obstetric outcome. Each year at age of birth and each kg/m^2^ of preconception BMI were associated with 5.1% (*P* = 0.001) and 3.2% (*P* = 0.036) higher odds of occurrence of an adverse obstetric outcome, respectively.

**Table 3:** Binary logistic regression for adverse obstetric outcomes with respect to all pregnancies.

Variables	OR	95% CI	*P* value
Iodine supplementation	0.748	0.557–1.006	0.055
Previous miscarriages	1.302	0.923–1.836	0.133
Maternal age (years)	1.049	1.018–1.080	0.001
High school (10–12 years)	1.024	0.708–1.480	0.901
Certificate after 12th grade	0.833	0.578–1.201	0.328
Parity (≥1 deliveries)	0.812	0.601–1.097	0.174
Smoking	1.111	0.772–1.599	0.569
Alcohol consumption	1.326	0.838–2.098	0.228
Thyroid disease	0.787	0.476–1.302	0.351
Preconception BMI (kg/m^2^)	1.032	1.002–1.063	0.036
Iron supplementation	0.128	0.082–0.199	<0.001

Comparators: no iodine supplementation, no miscarriage, ≤9 years of education, no smoking, no previous deliveries, no alcohol consumption, no thyroid disease, no iron supplementation.

OR, odds ratio.

We next searched for the factors influencing the number of obstetric outcomes (Table 4). Iodine intake was protective, with a 21% lower odds for the count of adverse obstetric outcomes, as was taking iron supplementation (52%, *P* < 0.001) and having had children (19%, *P* = 0.041). Age and BMI were positively associated: for every increase in age or in BMI the odds increased 3.3 and 2.3%, respectively, for the count of adverse obstetric outcomes.

**Table 4:** Poisson regression for the number of adverse obstetric outcomes with respect to all pregnancies.

Variable	OR	95% CI	*P* value
Iodine supplementation	0.791	0.651–0.960	0.018
Previous miscarriages	1.206	0.969–1.501	0.093
Maternal age (years)	1.033	1.014–1.053	<0.001
High school (10–12 years)	1.004	0.786–1.282	0.975
Certificate after 12th grade	0.914	0.716–1.167	0.470
Parity (≥1 deliveries)	0.812	0.666–0.991	0.041
Smoking	1.134	0.900–1.430	0.286
Alcohol consumption	1.018	0.757–1.369	0.907
Thyroid disease	0.881	0.639–1.215	0.441
Preconception BMI (kg/m^2^)	1.023	1.004–1.043	0.017
Iron supplementation	0.484	0.389–0.603	<0.001

Comparators: no iodine supplementation, no miscarriage, ≤9 years of education, no smoking, no previous deliveries, no alcohol consumption, no thyroid disease, no iron supplementation.

OR, odds ratio.

Table 5 reports the mode of delivery, newborn characteristics at birth and neonatal morbidities. Taking iodine supplements was associated with lower number of cesarean deliveries (small effect size; Pearson’s ϕ = 0.065). Even though well within the presently established normal reference range, newborn thyroid-stimulating hormone (TSH) levels were higher in newborns from women with iodine supplementation (small effect size, *r* = 0.067). Neonatal morbidities, birth weight, Apgar index, newborn cephalic perimeter and length, did not differ between groups. Additional comparisons are given in Supplementary Table 3.

**Table 5:** Mode of delivery, newborn characteristics and neonatal morbidities.

	No iodine, *n* = 479^a^	With iodine, *n* = 1162^a^	*P* value
Cesarean delivery (%)	35	28	0.009
Newborn characteristics			
Apgar index (1 min) ≤6 (%)	3	2	0.383
Birth weight (g; mean ± s.d.)	3175 ± 505	3226 ± 496	0.067
Cephalic perimeter (cm) (median; IQR)	34.5 (33.5–35.4)	34.5 (33.5–35.0)	0.370
Length (cm) (median; IQR)	48.5 (47.0–50.0)	48.5 (47.0–50.0)	0.513
TSH (mU/L) (median; IQR)	1.185 (0.680–1.910)	1.325 (0.800–2.043)	0.007
Neonatal morbidities (%)	8	6	0.137

^a^No information on: type of delivery (for 7 women with no iodine supplementation and for 1 woman with iodine supplementation), Apgar index (1 min) ≤6 (for 12 women with no iodine supplementation and for 9 women with iodine supplementation), birth weight (for 19 women with no iodine supplementation and for 27 women with iodine supplementation), cephalic perimeter (for 82 women with no iodine supplementation and for 66 women with iodine supplementation), length (for 80 women with no iodine supplementation and for 66 women with iodine supplementation), newborn TSH (for 19 women with no iodine supplementation and for 12 women with iodine supplementation).

IQR, interquartile range; TSH, thyroid-stimulating hormone.

Given the number of confounding variables to consider, we next performed the binary regression for neonatal morbidities. Intake of iodine and parity were associated with 47 (*P* = 0.024) and 57% (*P* = 0.011) lower odds of having neonatal morbidities, respectively (Table 6).

**Table 6:** Binary logistic regression for neonatal morbidities with respect to all viable deliveries.

Variables	OR	95% CI	*P* value
Iodine supplementation	0.528	0.303–0.920	0.024
Previous miscarriages	0.865	0.410–1.825	0.703
Maternal age (years)	1.031	0.972–1.095	0.309
High school (10–12 years)	1.228	0.565–2.669	0.605
Certificate after 12th grade	1.021	0.469–2.223	0.958
Parity (≥1 deliveries)	0.430	0.225–0.824	0.011
Smoking	1.261	0.624–2.548	0.518
Alcohol consumption	0.940	0.357–2.479	0.901
Thyroid disease	1.202	0.486–2.977	0.690
Preconception BMI (kg/m^2^)	1.003	0.943–1.068	0.917
Iron supplementation	0.802	0.235–2.731	0.724

Comparators: no iodine supplementation, no miscarriage, ≤9 years of education, no smoking, no previous deliveries, no alcohol consumption, no thyroid disease, no iron supplementation.

OR, odds ratio.

## Discussion

This study shows strong compliance to the recommendation (not mandatory) to supplement women with iodine during preconception, pregnancy and lactation, issued in 2013 by the NHA (15). The proportion of women with reported iodine supplementation increased from 25% in 2013 to 81% between 2014 and 2017. Iodine supplementation was mostly from iodine-containing multivitamin formula BR and from potassium iodide (approved by the authorities in March 2012) AR. It is important to mention that a recommendation is not mandatory, and clinicians may decide not to supplement for various reasons. Of notice, monitoring thyroid function is not included in current guidelines for pregnancy follow-up, except when thyroid disease is present (28).

Clinical records of iodine supplementation during breastfeeding were scarce, which may reflect undervaluation by the clinicians that iodine supplementation must continue during lactation.

Of the women taking iodine, 100% also reported folic acid intake and 93% also reported the use of iron supplements; while these numbers were only of 84 and 71%, respectively, for those not mentioning iodine supplementation.

In our study, when controlling for the confounding variables, iodine supplementation was associated with lower occurrence of adverse obstetric outcomes and neonatal morbidities. Fewer spontaneous miscarriages were observed in the supplemented group, with an increase AR in both groups, probably due to a more accurate registration in the clinical files over time. This observation adds to the literature, where data is scarce and contradictory. Gowachirapant *et al.* and Zhou *et al.* (29, 30) suggested that iodine supplementation (200 µg/day) in mildly iodine-deficient pregnant women had no benefit on newborn outcomes. A recent meta-analysis in euthyroid women showed that the pooled prevalence of preterm birth, low birth weight and hypertensive disorders were not associated with first trimester urinary iodine levels (31). Another systematic review and meta-analysis suggested that even though pre-eclamptic women presented lower urinary iodine levels, additional studies are needed to conclude on the association with iodine deficiency (32). Interestingly, in a Norwegian cohort study, iodine supplement use initiated before pregnancy reduced the risk of preeclampsia but not that of other measured adverse outcomes; this study did not address miscarriages (33). In the retrospective study presented here we had no information on urinary iodine or on the timing of iodine supplementation, to further associate the observed findings.

The overall impact of iodine supplementation in improving of maternal health should be viewed with caution within the general guidelines for pregnancy surveillance. The indicators from the Portuguese national low-risk pregnancy surveillance program showed an improvement after 2015 (34), namely, earlier surveillance and more adequate follow-up in pregnancy and puerperium (6 or more consultations), which may be related or independent from the 2013 recommendation on iodine supplementation. Pregnant women compliant with iodine supplementation may be more compliant with the pregnancy surveillance program, further contributing to the reduction in obstetric and neonatal complications.

Another important aspect relates with the dietary habits. This is particularly relevant since increased iodine intake during pregnancy may result from changes in the consumption of iodine-rich foods. In Portugal, despite the liberalization of iodized salt in 1996 and the implementation of measures for the use of iodized salt in school canteens, compliance has been scarce, and the use of dairy products remains the major source of iodine mainly in younger school-age population (silent prophylaxis) (35). Of interest, Japanese food became popular in the country in recent years, which is accompanied by increased consumption of iodine-rich algae. Additional detailed studies on dietary habits are needed to unravel whether food and/or intake of supplements influence the course of pregnancy and its obstetric and neonatal outcomes.

We observed that newborn TSH levels were within the normal reference ranges, still higher in the supplemented group, but the effect size was small. This observation warrants that additional longitudinal studies are necessary to infer on the biological significance of such finding and on its relationship with dosage and/or timing of initiation of iodine supplements intake. The incidence of congenital hypothyroidism in Portugal is of 1:2892 newborns and we observed only two cases of suspected congenital hypothyroidism in our sample, both AR (16). An Italian randomized single-blind and placebo-controlled trial with 90 women showed no differences on newborns' TSH levels between the two groups (36). In a recent meta-analysis, most studies showed no effect of iodine supplementation on maternal or infant TSH and free thyroxine serum levels (37). Nazeri *et al.* in a systematic review of the trials of the last 3 decades, showed no differences in weight, length or head circumference at birth, as we also observed here (38).

A final note on the comparison of the groups BR and AR. Women on iodine supplementation seem more compliant with pregnancy surveillance and recommendations, irrespectively of time.

There are meaningful limitations of this study. First, this is a retrospective observational study, so no causal relations can be established. Reporting bias is the main limitation. This may be particularly relevant before 2013 with respect to the registration of use of multivitamin supplements. If more women took iodine-containing vitamins without reporting and were, as such, included in the non-supplemented group, this may have diluted the observed effects. Also, information on the use of iodine supplements does not necessarily mean compliance. If less women then registered did comply with the recommendation, the observed differences may as well be lower than the reality. In both situations, the reported associations between iodine intake and adverse obstetric outcomes may be even higher than the observed.

Iodine supplementation is not necessarily synonymous of iodine sufficiency. We have no information about urinary iodine concentration, serum thyroid hormone levels or dietary iodine consumption for most of the women in the present study. Of interest, a recent study in the Portuguese São Miguel Azorean Island showed that the proportion of pregnant women with insufficient urinary iodine decreased from 99% BR to 91% AR. Despite of the amelioration, the data suggest that compliance with the iodine supplement intake is low (39).

Most clinical records did not include the precise timing of iodine supplementation, which may impact on the obstetric and neonatal outcomes. Additional studies are needed to understand whether and how the start time of iodine supplementation, and the women’s iodine nutritional status influence obstetric, neonatal and child development outcomes (40).

## Supplementary Material

Supplementary Table 1-Participant’s characterization. Additional comparisons

Supplementary Table 2-Adverse obstetric outcomes. Additional comparisons

Supplementary Table 3- Mode of delivery, newborn characteristics and neonatal morbidities. Additional comparisons

## Declaration of interest

The authors declare that there is no conflict of interest that could be perceived as prejudicing the impartiality of the research reported. T I M K has received lectureship fees from Merck, Goodlife Healthcare, Berlin Chemie and Quidell.

## Funding

This work has been funded by National funds, through the Foundation for Science and Technology (FCT) – project UIDB/50026/2020 and UIDP/50026/2020 and by the project NORTE-01-0145-FEDER-000039, supported by Norte Portugal Regional Operational Programme (NORTE 2020), under the PORTUGAL 2020 Partnership Agreement, through the European Regional Development Fund (ERDF). Funding agencies did not participate in any part of the research or in data or manuscript preparation.

## Statement of ethics

The study was authorized by national (National Data Protection Commission (authorization No. 11200/2017), regional (ACES Cávado I, Regional Health Administration of the North Committee on Health Ethics, Opinion no. 65/2018) and local (Braga’s Hospital Ethics Committee Ref. 16/2018) authorities and required no written informed consent.

## Data availability statement

The datasets used and/or analyzed during the current study will be available from the corresponding author on reasonable request.

## Author contribution statement

M L P, J A P, L V, M C N, P C, S R and T I M K contributed to the study design. N C S, J A P and M L P prepared the documentation for all ethical approvals. M L P, I C, A Q, A G and S R collected data. M L P, J A P and P C analyzed the data that was discussed with all authors. A G and S R prepared the figures. M L P and J A P wrote the first draft of the manuscript which was critically reviewed and approved in its final format by all authors. All the authors approved the final version to be published and agreed to be accountable for all aspects of the work in ensuring that questions related to the accuracy or integrity of any part of the work are appropriately investigated and resolved.
