# Supplementary material for: Iodine supplementation: compliance and association with adverse obstetric and neonatal outcomes
Source: Eur Thyroid J. 2021 Sep 16;11(1):e210035. doi: 10.1530/ETJ-21-0035 (PMC9142800; doi:10.1530/ETJ-21-0035)
Supplement: Supplementary Table 1-Participant’s characterization. Additional comparisons [file supplementary_table_1.pdf]

Supplementary Table 1-Participant's characterization. Additional comparisons

	**Before recommendation**			**After recommendation**			**Comparisons (*p* value)**				
	A1<br>No iodine<br>n=417#	B1<br>With iodine<br>n=138#	C1<br>Total<br>n=555	A2<br>No iodine<br>n=270#	B2<br>With iodine<br>n=1155#	C2<br>Total<br>n=1425	A1 α B1	A2 α B2	A1 α A2	B1 α B2	C1 α C2
Maternal age (y; mean±SD)	31 ± 6	32 ± 6	31 ± 6	34 ± 5	33 ±5	33 ± 5	0.203	0.004	< 0.001	0.305	< 0.001
Education (%)<br>≤9 y<br>High school (10-12 y)<br>Certificate after 12th grade	 27<br>33<br>40	 25<br>25<br>50	 26<br>31<br>42	 14<br>33<br>53	 18<br>28<br>53	 18<br>29<br>53	0.139	0.237	<0.001	0.285	<0.001
Parity (%)<br>Nullipara<br>Multiparous (≥1)	 52<br>48	 59<br>41	 54<br>46	 43<br>57	 52<br>48	 50<br>50	0.120	0.013	0.026	0.079	0.133
Previous miscarriages (%)	19	17	19	23	20	20	0.597	0.284	0.305	0.507	0.487
Thyroid disease (%)	7	3	6	23	7	10	0.090	< 0.001	<0.001	0.091	0.008
Smoking (%)	21	16	20	19	15	15	0.260	0.098	0.657	0.610	0.027
Alcohol consumption (%)	12	5	10	13	7	8	0.030	0.002	0.574	0.496	0.115
Folic acid supplementation (%)	84	100	88	84	100	98	<0.001	<0.001	0.853	NA	<0.001
Iron supplementation (%)	72	98	79	67	92	89	<0.001	<0.001	0.257	0.022	<0.001
Previous obesity (%)	10	9	10	22	17	18	0.560	0.184	<0.001	0.014	<0.001
Preconception BMI (Kg/m²; mean ± SD)	24.1 ±4.4	23.7 ± 3.8	24.0 ±4.3	24.3 ±4.8	24.2 ± 4.5	24.2 ± 4.6	0.395	0.668	0.546	0.266	0.380
Weight gain (kg; mean ± SD)	12.9 ± 4.9	13.0 ± 4.6	13.0 ± 4.8	11.3 ± 4.5	12.5 ± 5.0	12.4 ± 5.0	0.865	0.021	0.004	0.325	0.060

#No information: education [for 78 women before recommendation (54 without and 24 with iodine supplementation) and for 265 women after recommendation (65 without and 200 with iodine supplementation)], parity [for 5 women after the recommendation (1 without and 4 with iodine supplementation)], previous miscarriages (for 1 woman after the recommendation with iodine supplementation), thyroid disease [for 5 women before recommendation without iodine supplementation and for 24 women after the recommendation (12 without and 12 with iodine supplementation), smoking [for 20 women before recommendation (17 without and 3 with iodine supplementation) and for 170 women after recommendation (67 without and 103 with iodine supplementation), alcohol consumption [for 14 women before recommendation (12 without and 2 with iodine supplementation) and for 195 women after recommendation (73 without and 122 with iodine supplementation)], folic acid supplementation [for 63 women before recommendation (58 without and 5 with iodine supplementation) and for 105 women after recommendation (82 without and 23 with iodine supplementation)], iron supplementation[for 76 before recommendation (72 without and 4 with iodine supplementation) and for 250

women after recommendation (121 without and 129 with iodine supplementation)], obesity [for 4 women before recommendation without iodine supplementation and for 698 women after the recommendation (163 without and 535 with iodine supplementation)], preconception BMI [for 41 women before recommendation (33 without and 8 with iodine supplementation) and for 296 women after recommendation (109 without and 187 with iodine supplementation)], weight gain [for 211 before recommendation (179 without and 32 with iodine supplementation) and for 589 after recommendation (164 without and 425 with iodine supplementation)]. NA-Not applicable.
