# Supplementary material for: Iodine supplementation: compliance and association with adverse obstetric and neonatal outcomes
Source: Eur Thyroid J. 2021 Sep 16;11(1):e210035. doi: 10.1530/ETJ-21-0035 (PMC9142800; doi:10.1530/ETJ-21-0035)
Supplement: Supplementary Table 2-Adverse obstetric outcomes. Additional comparisons [file supplementary_table_2.pdf]

Supplementary Table 2-Adverse obstetric outcomes. Additional comparisons

	**Before recommendation**			**After recommendation**			**Comparisons (*p* value)**				
**All pregnancies**	A1<br>No iodine<br>n=417[#]	B1<br>With iodine<br>n=138#	C1<br>Total<br>n=555	A2<br>No iodine<br>n=270[#]	B2<br>With iodine<br>n=1155[#]	C2<br>Total<br>n=1425	A1 α B1	A2 α B2	A1 α A2	B1 α B2	C1 α C2
Adverse obstetric outcomes (≥1) (%)	46	40	44	57	32	37	0.242	< 0.001	0.002	0.074	0.004
Miscarriages (%)	7	2	6	30	9	13	0.031	< 0.001	< 0.001	0.005	< 0.001
Stillbirths (%)	0	0	0	0	0.4	0.4	NA	0.590 *	NA	>0.999*	0.330*
	**Before recommendation**			**After recommendation**			**Comparisons (*p* value)**				
**Viable pregnancies**	A1<br>No iodine<br>n=322[#]	B1<br>With Iodine<br>n=131	C1<br>Total<br>n=453	A2<br>No iodine<br>n=157	B2<br>With iodine<br>n=1031[#]	C2<br>Total<br>n=1188	A1 α B1	A2 α B2	A1 α A2	B1 α B2	C1 α C2
Fetal malformations (%)	2	2	2	1	1	1	>0.999*	0.286*	>0.999*	0.225*	0.052
Gestational diabetes (%)	7	12	8	17	11	12	0.104	0.051	<0.001	0.919	0.031
Gestational hypertension (%)	2	5	3	3	1	1	0.212*	0.265*	0.756*	0.014*	0.052
Hydramnios (%)	0	0.8	0.2	0.6	0.8	0.8	0.289*	>0.999*	0.328*	>0.999*	0.301*
Intrauterine growth restriction (%)	5	3	4	1	4	4	0.440	0.024	0.021	0.482	0.765
Large for gestational age	3	2	2	3	3	3	>0.999*	0.795*	0.766*	>0.999*	0.644
Placental abruption (%)	0	0	0	0	1	0.4	NA	>0.999*	NA	>0.999*	0.331*
Preeclampsia (%)	1	2	1	1	1	1	0.148 *	>0.999*	>0.999*	0.118*	0.549*
Preterm delivery (%)	6	6	6	3	3	3	0.967	0.991	0.162	0.124*	0.006
Risk of preterm delivery (%)	3	3	3	3	3	3	>0.999*	>0.999*	>0.999*	0.782*	0.734
Small for gestational age (%)	13	13	13	11	14	14	0.948	0.267	0.489	0.689	0.713

#No information for 7 newborns with respect to size for gestational age. *Fisher's exact test. NA = not applicable. Adverse obstetric outcomes (miscarriages, stillbirth, fetal malformations, gestational diabetes, gestational hypertension, hydramnios, intrauterine growth restriction, large for gestational age, placental abruption, preeclampsia, preterm delivery, risk of preterm delivery, small for gestational age).
