# Supplementary material for: Iodine supplementation: compliance and association with adverse obstetric and neonatal outcomes
Source: Eur Thyroid J. 2021 Sep 16;11(1):e210035. doi: 10.1530/ETJ-21-0035 (PMC9142800; doi:10.1530/ETJ-21-0035)
Supplement: Supplementary Table 3- Mode of delivery, newborn characteristics and neonatal morbidities. Additional comparisons [file supplementary_table_3.pdf]

Supplementary Table 3- Mode of delivery, newborn characteristics and neonatal morbidities. Additional comparisons

	Before recommendation			After recommendation			Comparisons (*p* value)				
	A1 No iodine n=322#	B1 With iodine n=131	C1 Total n=453	A2 No iodine n=157	B2 With iodine n=1031#	C2 Total n=1188	A1 α B1	A2 α B2	A1 α A2	B1 α B2	C1 α C2
Cesarean delivery (%)	34	29	32	36	28	29	0.259	0.047	0.707	0.900	0.178
**Newborn characteristics**											
Apgar index (1 min) ≤ 6 (%)	4	1	3	2	2	2	0.194*	>0.999*	0.564	0.349	0.717
Birth weight (g; mean ± SD)	3166±537	3157±422	3163±506	3193±431	3234±504	3229±496	0.856	0.347	0.598	0.094	0.019
Cephalic perimeter (cm) (median; IQR)	34.5 (33.5-35.3)	34.0 (33.2-35.0)	34.3 (33.3-35.2)	34.5 (33.5-35.5)	34.5 (33.5-35.0)	34.5 (33.5-35.1)	0.031	0.286	0.329	0.008	0.108
Length (cm) (median; IQR)	49 (47-50)	49 (48-50)	49 (47-50)	48.2 (47-50)	48.3 (47-50)	48.3 (47-50)	0.840	0.440	0.050	0.145	0.020
TSH (uUI/mL) (median; IQR)	1.045 (0.620 1.730)	1.260 (0.690-1.890)	1.120 (0.640-1.800)	1.420 (0.882-2.037)	1.330 (0.810-2.070)	1.350 (0.820-2.070)	0.219	0.368	<0.001	0.097	< 0.001
Neonatal morbidities (%)	10	9	10	4	6	5	0.800	0.374	0.020	0.098	0.001

#No information on: mode of delivery [for 5 women before the recommendation (4 with no iodine supplementation and 1 women with iodine supplementation) and for 3 women after the recommendation without iodine supplementation]. Apgar index (1 min) ≤ 6 [for 8 women before recommendation (7 without iodine supplementation and 1 with iodine supplementation) and for 13 women after recommendation (5 without iodine supplementation and 8 with iodine supplementation)]. birth weight [for 10 women before recommendation (9 without iodine supplementation and 1 with iodine supplementation) and for 36 women after recommendation (10 without iodine supplementation and 26 with iodine supplementation). cephalic perimeter [for 100 women before recommendation (70 without iodine supplementation and 30 with iodine supplementation) and for 48 women after recommendation (12 without iodine supplementation and 36 with iodine supplementation). length [for 98 women before recommendation (68 without iodine supplementation and 30 with iodine supplementation) and for 48 women after recommendation (12 without iodine supplementation and 36 with iodine supplementation)]. newborn TSH [for 18 women before recommendation (14 without iodine supplementation and 4 with iodine supplementation) and for 13 women after recommendation (5 without iodine supplementation and 8 with iodine supplementation).

*Fisher's exact test. SD-standard deviation. IQR-interquartile range.
